# Plant-mediated RNAi silences midgut-expressed genes in congeneric lepidopteran insects in nature

**DOI:** 10.1186/s12870-017-1149-5

**Published:** 2017-11-13

**Authors:** Spoorthi Poreddy, Jiancai Li, Ian T. Baldwin

**Affiliations:** 1 Department of Molecular Ecology, Max-Planck-Institute for Chemical Ecology, Hans-Knöll-Str. 8, D-07745 Jena, Germany; 2 Present address: Department of Plant and Microbial Biology, University of Zurich, Zollikerstrasse 107, CH-8008 Zurich, Switzerland

**Keywords:** RNA interference, Plant-mediated RNAi, *Manduca quinquemaculata*, *CYP6B46*, *β-glucosidase*, Transgenic tobacco plants

## Abstract

**Background:**

Plant-mediated RNAi (PMRi) silencing of insect genes has enormous potential for crop protection, but whether it works robustly under field conditions, particularly with lepidopteran pests, remains controversial. Wild tobacco *Nicotiana attenuata* and cultivated tobacco (*N. tabacum*) (Solanaceae) is attacked by two closely related specialist herbivores *Manduca sexta* and *M. quinquemaculata* (Lepidoptera, Sphingidae)*.* When *M. sexta* larvae attack transgenic *N. attenuata* plants expressing double-stranded RNA(dsRNA) targeting *M. sexta’s* midgut-expressed genes, the nicotine-ingestion induced *cytochrome P450 monooxygenase* (invert repeat (ir)CYP6B46-plants) and the lyciumoside-IV-ingestion induced *β-glucosidase1* (irBG1-plants), these larval genes which are important for the larvae’s response to ingested host toxins, are strongly silenced.

**Results:**

Here we show that the PMRi procedure also silences the homologous genes in native *M. quinquemaculata* larvae feeding on irCYP6B46 and irBG1-transgenic *N. attenuata* plants in nature. The PMRi lines shared 98 and 96% sequence similarity with *M. quinquemaculata* homologous coding sequences, and CYP6B46 and BG1 transcripts were reduced by ca. 90 and 80%, without reducing the transcripts of the larvae’s most similar, potential off-target genes.

**Conclusions:**

We conclude that the PMRi procedure can robustly and specifically silence genes in native congeneric insects that share sufficient sequence similarity and with the careful selection of targets, might protect crops from attack by congeneric-groups of insect pests.

**Electronic supplementary material:**

The online version of this article (10.1186/s12870-017-1149-5) contains supplementary material, which is available to authorized users.

## Background

RNA interference (RNAi) is a sequence-specific, double stranded RNA (dsRNA) induced gene-silencing mechanism that operates at transcriptional or post-transcriptional levels and is conserved across all the eukaryotes [1], including lepidopteran insects [2]. Since the RNAi mechanism was first demonstrated in *Caenorhabditis elegans* in 1998, RNAi has emerged as a potent gene-silencing tool for loss-of-function analyses in a wide range of organisms [1, 3, 4]. RNAi has emerged as the most powerful technique to analyze gene function in insects, for which stable transgenesis is not available [2, 5]. The success of RNAi in insects is highly dependent on the insect-species, target gene and its function, organ of gene expression and mode of delivery of silencing molecules [2, 6]. RNAi-mediated gene silencing in some insect species is quite robust and can even be transmitted to subsequent generations via germ line transmission [7–9]. Coleopteran species are highly sensitive to RNAi and more efficient gene silencing can easily achievable in coleopteran insects when compared to insect species in other orders [10, 11]. In contrast, lepidopteran species, particularly in laboratory experiments with injected dsRNA are recalcitrant to the procedure [2, 12].

Although the silencing of target genes in insects is achieved by various dsRNA introduction strategies, such as microinjection or oral delivery via artificial diets [13], gene silencing can be enhanced and possibly achieved under field conditions by engineering host plants to produce dsRNAs [5]. An efficient method to down-regulate cotton bollworm defense genes using plant-mediated RNAi (PMRi) has been described by engineering host plants to produce dsRNAs directed against bollworm larvae’s P450 monooxygenase (CYP6AE14) gene in laboratory experiments [15]. Several studies have demonstrated successful gene silencing in various insect orders such as Coleoptera, Lepidotera and Hemiptera using stable or transient transgenic PMRi plants [14–21]. A recent advance in PMRi to control insects was achieved by expressing exogenous dsRNAs targeted against the β-actin gene of the Colorado potato beetle (CPB) via chloroplast transformation in potato. Transplastomic potato plants were shown to be lethal to CPB larvae and were protected from CPB attack in glasshouse experiments [22].

The capacity of insect pests to adapt to conventional insecticides [23] or to *Bacillus thuringiensis* (Bt) expressing plants [24] is an ongoing concern for the long-term pest management of crop plants. The PMRi approach offers the potential to develop insect-resistant crops that produce insecticidal dsRNAs, but only a few reports have demonstrated its potential under field conditions [17, 20, 25, 26]. Crop plants are commonly attacked by congeneric insect pests, for instance *Spodoptera litura* and *S. exigua* are both major pests of various crops [27]; *Helicoverpa armigera* and *H. punctigera* are major pests of cotton [28]; *H. armigera* and *H. zea* attack maize plants [29]. Hence whether PMRi can silence conserved essential genes in congeneric insect pests under field condition would be an important test of this procedures utility as a crop protection tool.

The congeneric species, tobacco hornworm [*Manduca sexta* (*Ms*)*,* Linnaeus, 1763] and tomato hornworm [*M. quinquemaculata* (*Mq*)*,* Haworth, 1803] (Lepidoptera: Sphingidae) are closely related sympatric sibling species [30–32] that both attack solanaceous crops in North America [33]. These two insects have similar morphologies [34], behavior [35], and ecology [36] and their host-plant interactions have been intensely studied [31, 37–39]. Both of these hornworm species are significant pests on solanaceous crops in North America. In nature, the annual tobacco plant *Nicotiana attenuata* Torr. ex Wats, native to Great Basin Desert of southwestern Utah is a common host plant for both of these pest insects and both also attack cultivated tobacco.

Recently, we elucidated *M. sexta’s* counter-adaptation strategies against two of *N. attenuata*’s major chemical defenses, namely nicotine and 17-hydroxygeranyllinalool diterpene glycosides (HGL-DTGs), by silencing *M. sexta*’s midgut-expressed *cytochrome P450 monooxygenase* (*CYP6B46*) and *β-glucosidase1* (*BG1*) genes. This was accomplished by developing transgenic *N. attenuata* PMRi lines that expressed inverted repeat (ir) constructs of CYP6B46 and BG1 and planting these irCYP6B46 and irBG1 plants into field plots in the plants and insects native habitat [25, 26]. Since *M. sexta* and *M. quinquemaculata* are closely related, co-occurring species in *N. attenuata*’s native habitat [40], we hypothesized that PMRi plants producing *M. sexta*’s CYP6B46 and BG1 dsRNA could silence the homologous genes in native *M. quinquemaculata*.

## Results

### Experimental system used to evaluate homologous gene silencing

**Fig. 1:**
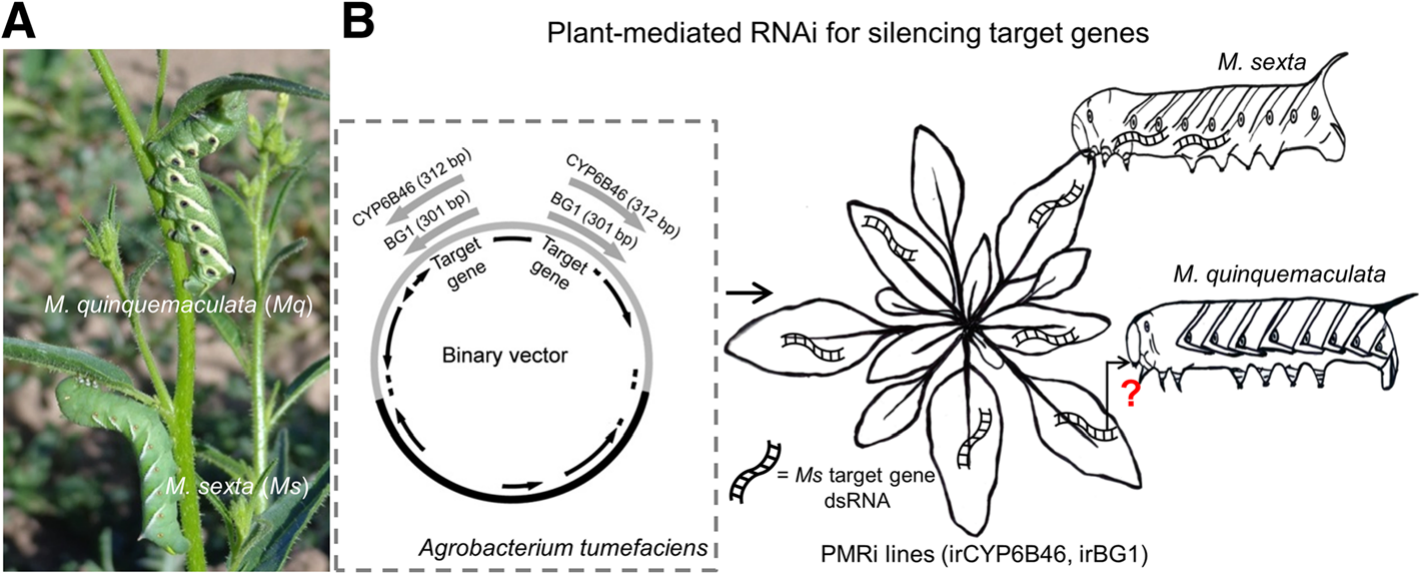
Experimental system used to evaluate if the PMRi lines generated from *Manduca sexta* genes can silence *M. quinquemaculata* homologous genes. **a** Fifth-instar *M. sexta* and its closely related species *M. quinquemaculata* larvae on their native host plant, the wild tobacco *Nicotiana attenuata* in Great Basin Desert of southwestern Utah. **b** Schematic overview of plant-mediated RNAi: the binary vector constructed to independently express ~300 bp dsRNA of *M. sexta*’s target genes such as *cytochrome P450 monooxygenase* (*CYP6B46*) and *β-glucosidase1* (*BG1*) in *N. attenuata.* The trophic transfer of these dsRNA from plant to *M. sexta* larvae silences their respective target gene expression. PMRi lines generated with *M. sexta* dsRNA were used to test if the trophic transfer can also silence homologous gene expression in *M. quinquemaculata* larvae in nature

*M. sexta* and *M. quinquemaculata* are two specialist herbivores of *N. attenuata* (Fig. 1a). Previously, we silenced the expression of *CYP6B46* and *BG1* in *M. sexta* larval midguts using PMRi lines irCYP6B46 [25] and irBG1 [26], respectively. These stable transgenic irCYP6B46 and irBG1 *N. attenuata* lines were generated using *Agrobacterium tumefaciens*-mediated transformation with an inverted repeat of 312 and 301 bp *Ms*CYP6B46 and *Ms*BG1 complementary DNA (cDNA), respectively. Here, we utilized these PMRi plants generated from *M. sexta* genes to examine the silencing of the homologous genes in a wild population of *M. quinquemaculata* (Fig. 1b).

### *M. sexta CYP6B46* and *BG1* genes share high sequence similarity with *M. quinquemaculata* homologous genes

**Fig. 2:**
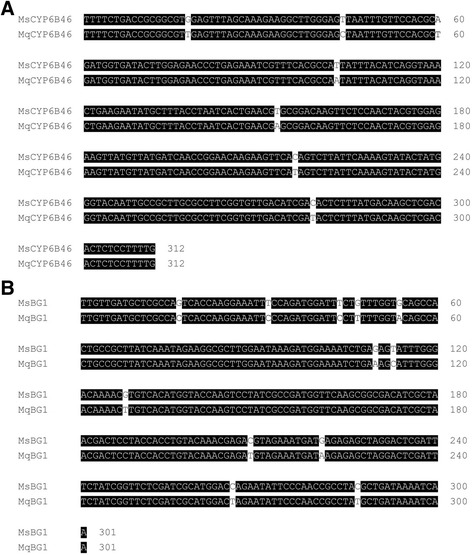
Alignments of *M. sexta* cDNA regions selected to generate PMRi lines with *M. quinquemaculata* homologs. **a** *M. sexta* CYP6B46 fragment cloned to generate inverted-repeat (ir) CYP6B46 showed 98.1% sequence similarity with *M. quinquemaculata* CYP6B46 and (**b**) *M. sexta* BG1 fragment cloned to generate irBG1 showed 96% similarity with *M. quinquemaculata* BG1. Identical homologous regions of >21 nt were identified in the alignment of *M. sexta* CYP6B46 and BG1 cDNA regions selected to generate PMRi lines with that of *M. quinquemaculata* CYP6B46 and BG1, respectively

Since PMRi operates in a sequence specific manner to silence target gene transcripts, homologous gene silencing depends on sequence similarities between homologous genes particularly in the regions that are used in the generation of the transgenic plants. To test the sequence similarities of *Ms*CYP6B46 and *Ms*BG1 with their corresponding homologous genes in *M. quinquemaculata*, we aligned the partial coding sequences of *Ms*CYP6B46 (312 bp) and *Ms*BG1 (301 bp) used in the generation of the irCY6B46 and irBG1 PMRi plants with the coding regions of *M. quinquemaculata’s* homologous genes. We found that the *M. sexta* CYP6B46 fragment cloned to generate the irCYP6B46 plants shared 98% sequence similarity with the *M. quinquemaculata* CYP6B46 sequence (Fig. 2a) and that the *M. sexta* BG1 fragment cloned to generate the irBG1 plants shared 96% sequence similarity with the *M. quinquemaculata* BG1 sequence (Fig. 2b). Notably, five identical homologous regions of >21 nt were identified in the alignment of *Ms*CYP6B46 and *Mq*CYP6B46. The lengths of these identical regions were 25 nt (+19 to 43), 42 nt (+61 to 102), 49 nt (+104 to 152), 62 nt (+154 to 215), 62 nt (+217 to 278), 33 nt (+280 to 312) (Fig. 2a). Three identical homologous regions of >21 nt were identified in the alignment of *Ms*BG1 and *Mq*BG1. The lengths of these identical regions were 55 nt (+55 to 109), 80 nt (+129 to 208), 44 nt (+222 to 265) (Fig. 2b).

### Silencing of *CYP6B46* and *BG1* in wild *M. quinquemaculata*

**Fig. 3:**
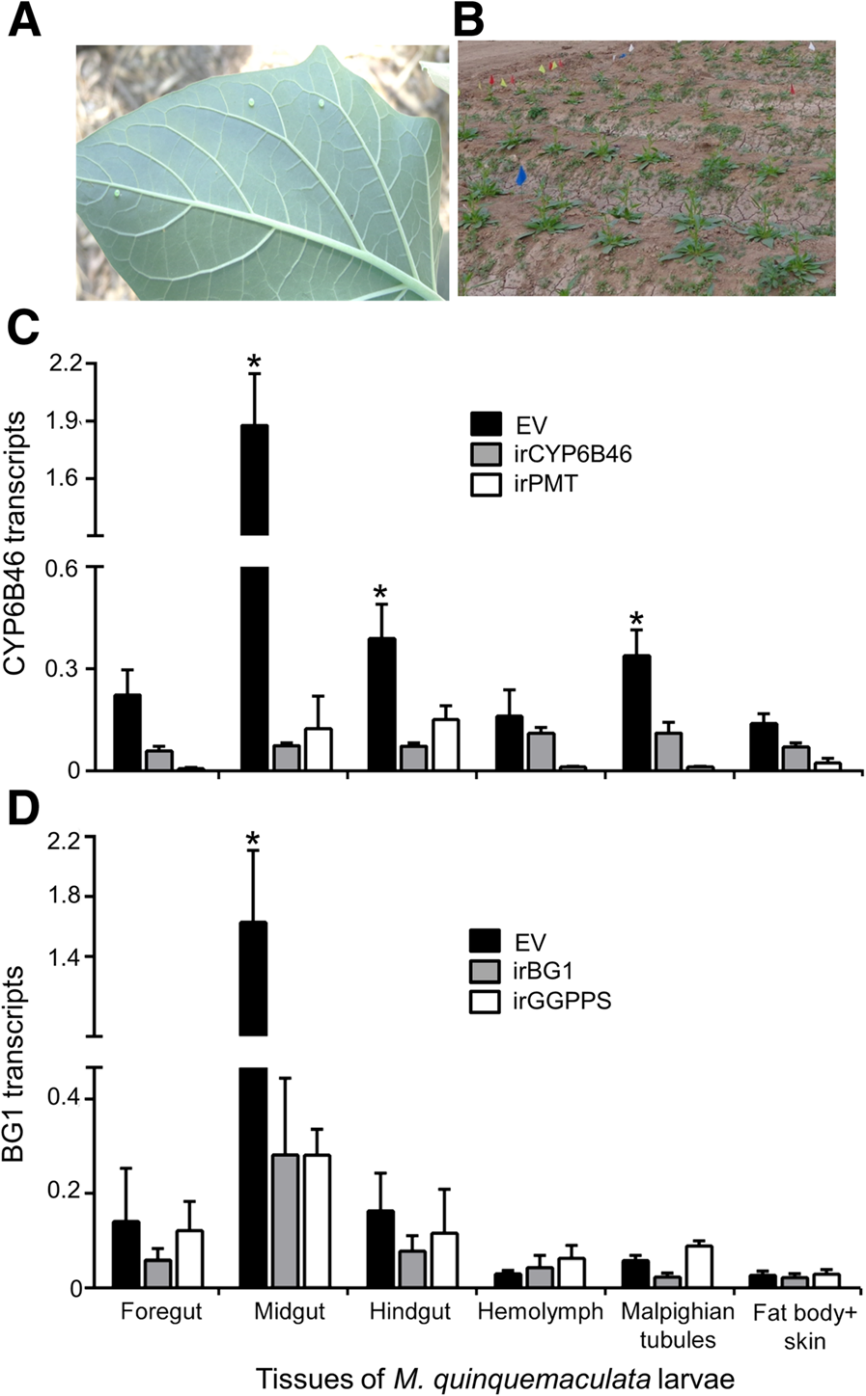
Silencing of midgut-expressed *CYP6B46* and *BG1* genes in wild *M. quinquemaculata* larvae feeding on WT, PMRi, and nicotine and DTG-depleted plants. **a** Wild *Manduca spp.* eggs oviposited on *Datura wrightii* provided the source of *M. quinquemaculata* larvae which can be distinguished from *M. sexta* larvae when the larvae reach the third- instar. **b** PMRi *N. attenuata* lines and *N. attenuata* plants transformed by RNAi to silence: nicotine biosynthesis, by expressing an inverted repeat (ir) construct of the host plant’s *putrescine N-methyl transferase* (irPMT) and 17-hydroxygeranyllinalool diterpene glycoside (HGL-DTGs) biosynthesis, by expressing an ir construct of *geranylgeranyl pyrophosphate synthase* (irGGPPS); planted in a field plot in Great Basin Desert of southwestern Utah. **c** *M. quinquemaculata* CYP6B46 transcripts (relative to ubiquitin) in various tissues of fourth-instar larvae fed on EV, irCYP6B46 and irPMT plants (midgut: *F*_*2,15*_ = 7.219 *P* ≤ 0.006; hindgut: *F*_*2,15*_ = 6.651 *P* ≤ 0.008; Malpighian tubules: *F*_*2,15*_ = 10.604 *P* ≤ 0.001; *n* = 6 in all bars). Note that feeding on nicotine-containing WT plants strongly induces the midgut expression of CYP6B46 transcripts and that feeding on the PMRi plants which contain WT levels of nicotine deplete CYP6B46 transcript abundance to levels found in larvae feeding on nicotine-depleted irPMT plants. **d** *M. quinquemaculata* BG1 transcripts (relative to ubiquitin) in various tissues of fourth-instar larvae feeding on EV, irBG1 and irGGPPS plants (midgut: *F*_*2,14*_ = 9.458 *P ≤* 0.002; n = 6 in EV, GGPPS and 5 in BG1 group). Note that feeding on HGL-DTG-containing WT plants strongly induces the midgut expression of BG1 transcripts and that feeding on the PMRi plants which contain WT levels of HGL-DTGs deplete BG1 transcript abundance to levels found in larvae feeding on HGL-DTG-depleted irGGPPS plants. Asterisks indicate significant differences between means (± SE) in comparison to EV, determined by one-way ANOVA and Fisher LSD post hoc, which was conducted separately for each tissue

Naturally oviposited wild *Manduca spp.* eggs (Fig. 3a) were collected from the study area and larvae were reared on empty vector (EV), inverted repeat *putrescine N-methyl transferase* (irPMT), inverted repeat *geranylgeranyl pyrophosphate synthase* (irGGPPS), irCYPB46 and irBG1 *N. attenuata* plants (Table 1) that were planted in a field plot located in *N. attenuata*’s native habitat (Fig. 3b), until they had reached the fourth-instar. The two species are easily distinguished when they attain the third-instar. In *M. sexta* larvae CYP6B46 and BG1 are the midgut-expressed genes that are induced in response to the ingestion of nicotine and lyciumoside IV (a major HGL-DTG), respectively. To test whether these candidate genes were midgut expressed in *M. quinquemaculata,* and are induced by the ingestion of nicotine and lyciumoside IV, we profiled their transcript levels in various tissues, including foregut, midgut, hindgut, hemolymph, Malpighian tubules and fat body along with skin, in nicotine and lyciumoside IV containing EV-fed control, nicotine depleted irPMT-fed, and lyciumoside IV depleted irGGPPS-fed larvae. Both the genes, namely CYP6B46 (*P* ≤ 0.001; Fig. 3c) and BG1 (P ≤ 0.001; 3D) were found to have relatively higher expression levels in the midguts of larvae feeding on EV plants than in the larvae feeding on irPMT and irGGPPS plants, respectively suggesting that feeding on nicotine- and lyciumoside IV-containing EV plants strongly induced the midgut expression of CYP6B46 and BG1 transcripts. Although the transcript abundance of CYP6B46 was higher (*P* ≤ 0.005; Fig. 3c) in hindguts and Malpighian tubules of larvae feeding on EV plants than in the larvae feeding on irPMT plants, the CYP6B46 expression in these tissues is significantly lower (six-fold) as compared to that of midguts.

**Table 1:** APHIS notification numbers for importing seeds and releasing transgenic *N. attenuata* plants

Line	Import #	Year	Release #
EV	07–341-101n	2014	13–350-101r
irPMT (*NaPMT* NCBI accession no. AF280402)	07–341-101n	2014	13–350-101r
irGGPPS (*NaGGPPS* NCBI accession no. EF382626)	07–341-101n	2014	13–350-101r
irCYP6B46 (*MsCYP6B46* NCBI accession no. GU731529)	10–004-105 m	2014	13–350-101r
irBG1 (*MsBG1* NCBI accession no. FK816842)	10–004-105 m	2014	13–350-101r

Midguts of irCYP6B46-fed larvae showed >90% lower abundance of CYP6B46 transcripts than that of EV-fed larvae. Feeding on the irCYP6B46 PMRi plants which contain nicotine levels similar to those of EV plants reduced the CYP6B46 transcript levels to that found in larvae feeding on nicotine-depleted irPMT plants (Fig. 3c; *F*_*2,15*_ = 7.219 *P* ≤ 0.006). Transcript abundance of BG1 in the midguts of irBG1-fed larvae was >80% lower than that of EV-fed larvae and the reduction in BG1 transcripts was not observed in other tissues of irBG1-fed larvae. Feeding on the irBG1 PMRi plants which contain HGL-DTGs similar to those of EV plants reduced the BG1 transcript abundance to levels found in larvae feeding on HGL-DTG-depleted irGGPPS plants (Fig. 3d; *F*_*2,14*_ = 9.458 *P ≤* 0.002).

### Sequence analysis of *MqCYP6B46* and *MqBG1* with their potential off-target genes

**Fig. 4:**
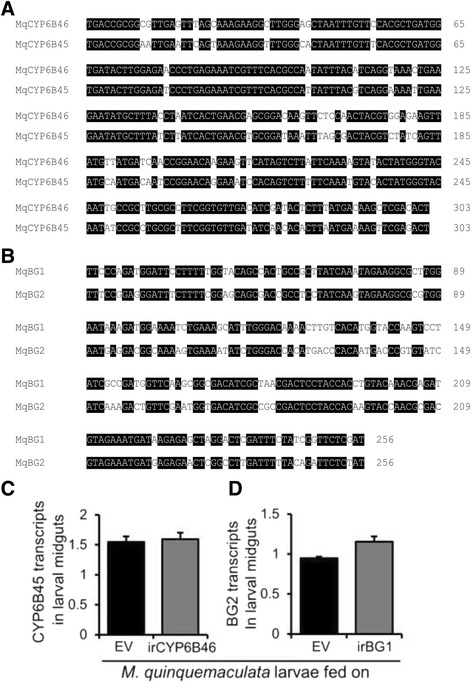
Silencing of *MqCYP6B46* and *MqBG1*does not silence the most closely related, likely non-target genes. **a** Alignment of *Mq*CYP6B46 partial coding sequence with *Mq*CYP6B45 showed 83% sequence similarity and one identical homologous region >21 nt was identified. **b** Alignment of *Mq*BG1 coding sequence with *Mq*BG2 showed 71% sequence similarity and no identical homologous regions >21 nt were identified. Transcripts (relative to ubiquitin) of (**c**) *MqCYP6B45* and (**d**) *MqBG2* in the midguts of fourth-instar EV-, irCYP6B46 and EV-, irBG1-feeding larvae did not show off-target silencing of CYP6B45 and BG2, respectively, (n = 6) Significant differences between means (± SE) in comparison to EV was determined by one-way ANOVA and Fisher LSD post hoc test

Gene silencing may also result in silencing of non-target genes that belong to closely related gene families if they share high sequence similarities in the targeted regions. Off-target effects could be minimized by using a region of coding sequence for inverted repeat construct preparation which is specific to the targeted gene and do not share high sequence similarity with other closely related genes. Sequence similarities of *Mq*CYP6B46 and *Mq*BG1 coding sequences with their corresponding potential off target genes *Mq*CYP6B45 and *Mq*BG2, respectively were determined by aligning their coding regions. *Mq*CYP6B46 partial coding sequence had 82% sequence similarity to *Mq*CYP6B45 and one identical homologous region with a continuous stretch >21 nt (Fig. 4a). The *Mq*BG1 partial coding sequence shared 71% sequence similarity with *Mq*BG2 and no identical homologous regions with a continuous stretch >21 nt (Fig. 4b). *Mq*CYP6B46 and *Mq*BG1 sequences didn’t show sequence similarity >21 nt with the sequences of other non-target organisms that are available in NCBI database.

### Silencing of *MqCYP6B46* and *MqBG1* is target gene specific

To determine whether silencing of *MqCYP6B46* and *MqBG1* was target-gene specific, we quantified the transcript levels of *Mq*CYP6B45 and *Mq*BG2, respectively. CYP6B45 transcript levels in the midguts of larvae feeding on irCYP6B46 were as not reduced in comparison to those of EV-fed larvae (Fig. 4c). From these results, we infer that the one identical continuous stretch >21 nt was not used in the production of potential small interfering RNA or that the silencing signal was not sufficient to reduce CYP6B45 transcript levels. Transcript abundance of BG2 in the midguts of larvae feeding on irBG1 plants was also not reduced and even slightly higher than those of EV-fed larvae (Fig. 4d). These results suggest that *Mq*CY6B46 and *Mq*BG1 silencing by PMRi was highly sequence-specific and free of off-target effects.

## Discussion

Silencing of insect genes in congeneric species in their native habitat by *in planta* expression of insecticidal dsRNAs holds a great promise for the future development of broad spectrum insect resistant crop plants [41, 42]. Previously, we showed that plant-mediated RNAi is highly efficient in silencing *M. sexta* genes in its natural environment [25, 26]. In this study, we showed that the homologous target genes in the midgut of the lepidopteran *M. quinquemaculata* can be suppressed by feeding native *M. quinquemaculata* larvae on transgenic *N. attenuata* plants producing dsRNA against *M. sexta* midgut-expressed genes, under field conditions. Although we did not collect the precise data on larval life cycle parameters and defoliation rates while feeding on transgenic plants, we did not observe any noticeable differences in larval mass and plant defoliation rates between those feeding on control (EV) and transgenic RNAi lines (irCYP6B46 and irBG1).

The RNAi-induced silencing in insects is initially achieved majorly via three dsRNA-delivery methods, such as injection [43], ingestion [14] and feeding dsRNA producing bacteria [44]. These delivery methods were not efficient in silencing of target genes in lepidoteran insects perhaps due to the instability of dsRNAs upon exposure to the nuclease rich environment of the larval gut [17, 19, 45]. However, the following reports successfully demonstrated gene silencing in *M. sexta,* firstly the integrin-beta1 gene silencing is achieved by injection of dsRNAs. The integrin-beta1 gene is required for haemocytic encapsulation and its silencing decreased the encapsulation of haemocytes [46]. In the other study, silencing of *M. sexta* nitric oxide synthase was reported [47]. RNAi-induced silencing in larval chemosensory tissues was achieved by Howlett and colleagues by feeding dsRNA targeting a chemosensory receptor gene in *M. sexta* [48].

In addition to the abovementioned successful RNAi-induced silencing studies in *M. sexta*, Terenius and colleagues have also reported the variable sensitivity of *M. sexta* larvae to RNAi techniques, particularly when dsRNA is delivered by injection [2]. The efficiency of target gene silencing may be enhanced by the continuous supply of dsRNA, because the core RNAi genes in *M. sexta* larvae are known to be induced in response to the dsRNA injection and elevated when contact with exogenous dsRNA is prolonged [12]. Injected dsRNA into *M. sexta* hemolymph is unstable and rapidly degraded by RNAse [49].

The PMRi approach, in which the insect’s host plant is utilized as an oral delivery vehicle of dsRNAs could circumvent the problem of dsRNA degradation by continuously supplying dsRNA to feeding larvae [14, 16]. *M. sexta* midgut-expressed, nicotine-ingestion induced *CYP4B46*, *CYP4M1* and *CYP4M3* genes are efficiently silenced without any co-silencing of closely related non target genes, using plant virus based dsRNA-producing system (VDPS) [19]. VDPS based target gene silencing is rapid and does not require the laborious generation of stable transgenic plants; however, this method is transient and cannot yet be used under field conditions. Whereas, silencing of *M. sexta* midgut genes *CYP6B46* [25] and *BG1* [26] was achieved under field conditions using PMRi approach and revealed the role of *CYP6B46* and *BG1* in *M. sexta*’s counter-adaptation strategies against *N. attenuata’s* defense metabolites nicotine and lyciumoside IV. Yet, the application of PMRi has certain limitations, mainly off-target effects [50], silencing of the genes in non-targeted organisms in the field conditions. The off-target effects can be circumvented by careful selection of target pest gene sequences and performing thorough bioinformatics analysis to determine whether they share potential sequence similarity with the homologous genes in the other organisms [41, 50, 51]. On the other hand, PMRi approach is a potential tool for silencing the target genes in the congeneric pest species if they share sufficient sequence similarity as showed in the present study.

## Conclusions

We showed that PMRi transgenic plants generated with *M. sexta* genes silences the *CYP6B46* and *BG1* homologous genes in native *M. quinquemaculata*. The PMRi lines shared 98 and 96% sequence similarity with *M. quinquemaculata* homologous coding sequences, and CYP6B46 and BG1 transcripts were reduced by ca. 90 and 80%, without any off-target effects. We conclude that the PMRi procedure can robustly and specifically silence genes in congeneric insects that share sufficient sequence similarity for the targeted genes when feeding on transformed plants growing in the rough and tumble of nature. An important implication of the work is that with the careful selection of targets, the PMRi procedure might be used to protect crops from attack by congeneric groups of insect pests.

## Methods

### Plant material

All the stable transgenic *N. attenuata* lines EV [52], irPMT [53], irGGPPS [54], irCYP6B46 [19] and irBG1 [26] used in the study were previously characterized. irPMT *N. attenuata* plants (A-03-108-3) were used as nicotine deficient host plants, irGGPPS *N. attenuata* plants (A-07-230-5) were used as HGL-DTG depleted host plants, irCYP6B46 transgenic *N. attenuata* PMRi plants (A-09-30-2) were used to silence *M. quinquemaculata* CYP6B46, irBG1 transgenic *N. attenuata* PMRi plants (A-08-375-10) were used to silence *M. quinquemaculata* BG1 and EV-transformed plants (A-04-266-3) were used as transgenic controls. Silencing of *M. sexta* CYP6B46 and BG1 using irCYP6B46 and irBG1 PMRi plants, respectively was reported previously [25, 26].

These transgenic lines were developed from *N. attenuata* 30× inbred seeds, which was originally collected in 1988 from a native population in Utah (USA) [55]. Field experiments were conducted at the Lytle Ranch Preserve in Santa Clara, Utah, 84,765 (37° 08′ 45″ N, 114° 01′ 11″W) in 2014. Seeds of *N. attenuata* EV, irPMT, irGGPPS, irCYP6B46 and irBG1 lines were imported and released in accordance with Animal and Plant Health Inspection Service notifications (Table 1). Seedlings of *N. attenuata* were transferred to 50 mm peat pellets (Jiffy) 15 days after germination, then seedlings were kept in the shade to get acclimatized to the environmental conditions of high sunlight and low relative humidity over 14 days. Adapted early rosette-stage plants were transplanted in to the field plot and plants were watered regularly until roots were established.

#### *M. quinquemaculata*

Wild *Manduca spp.* eggs were collected from natural ovipositions on native *N. attenuata* and *Datura* populations in the study area during May 2014. The hatched neonates were transferred to rosette-stage *N. attenuata* plants and reared until they had reached the third instar when the two species, the tobacco hornworm (*M. sexta*) and the tomato hornworm (*M. quinquemaculata*) can be morphologically distinguished. When the *M. quinquemaculata* larvae had reached the fourth-instar they were used in all the experiments.

### Harvesting larval tissues

Fourth-instar *M. quinquemaculata* larval tissues such as foregut, midgut, hindgut, hemolymph, Malpighian tubules and fat body along with skin were collected in RNA-later for transcript profiling of target and off-target genes. Larvae were immobilized by placing them on ice before dissection. First, haemolymph was collected by clipping the larval horn, as previously described [19]. Then, larvae were dissected to collect various tissues. Gut content and peritrophic membrane was removed before midgut collection. Dissected tissues were immediately transferred to RNA-later solution (Ambion) and stored at −80 °C as recommended by the manufacturer, until further use.

### Total RNA isolation and cDNA synthesis

Tissues stored in RNA later solution were recovered and total RNA was extracted using TRI reagent (Invitrogen) according to manufacturer’s protocol. The integrity and purity of extracted RNA was monitored by denaturing agarose gel electrophoresis and nanodrop spectrophotometer. Isolated total RNA was subjected to TURBO DNase (Ambion) treatment to remove genomic DNA contamination. For each sample, 500 ng of total RNA was used for cDNA synthesis using oligo(dT)18 primer and SuperScript II enzyme (Invitrogen) according to manufacturer’s instructions.

### Sequencing of partial coding regions

*M. quinquemaculata Ubiquitin*, *CYP6B46*, *CYP6B45*, *BG1* and *BG2* gene sequences were initially retrieved from *M. quinquemaculata* midgut transcriptome and partial coding sequences of all these genes were sequenced by Sanger dideoxy sequencing method. The obtained partial coding sequences of *Ubiquitin*, *CYP6B46*, *CYP6B45*, *BG1* and *BG2* were submitted to NCBI under the accession numbers KX074011, KX074015, KX074014, KX074013 and KX074012, respectively. The primer sequences used for amplification of partial coding sequences were listed in Table 2.

**Table 2:** *M. quinquemaculata* gene primers used in various experiments

No	Gene	Primer sequences (5′-3′)	Use
1	*MqUbiquitin*	For- CAAGAAGCGCAAGAAGAAGAAC	Internal control for *M. quinquemaculata* transcript quantification
		Rev- CGTCCACCTTGTAGAACCTAAG	
2	*MqBG1*	For- CCAACCGCCTATGCTGATAAA	Transcript quantification and testing the silencing efficiency of *M. quinquemaculata BG1*
		Rev- GTGACCATGGGTTGGATGTT	
3	*MqBG2*	For- GCTGTATGTTACGGCCAAGA	Transcript quantification and testing the co-silencing efficiency of *M. quinquemaculata BG2*
		Rev- CACGCGCCTTCTACTTGATA	
4	*MqCYP6B46*	For- GTGCCTATTACTCCGCGATCTA	Transcript quantification and testing the silencing efficiency of *M. quinquemaculata CYP6B46*
		Rev- CAAGCCTTCTTTGCTAAACTCC	
5	*MqCYP6B45*	For- GAAATGGATAAATTGGTTTTGACC	Transcript quantification and testing the co-silencing efficiency of *M. quinquemaculata CYP6B45*
		Rev- TTATTTTGACAGAGAAGATTGAGG	
6	*MqUbiquitin*	For- CGACTACAACATCCAGAAGGAG	Amplification of partial coding sequence of *M. quinquemaculata Ubiquitin*
		Rev- GGCTTACGGCTACATCTTAGTC	
7	*MqBG1*	For- GAAGTTGTTGATGCTCGCC	Amplification of partial coding sequence of *M. quinquemaculata BG1*
		Rev- GTGACCATGGGTTGGATG	
8	*MqBG2*	For- GCTGTATGTTACGGCCAAG	Amplification of partial coding sequence of *M. quinquemaculata BG2*
		Rev- GGCTGAATATTGTATTTAAGC	
9	*MqCYP6B46*	For- TGCCTATTACTCCGCGATCTA	Amplification of partial coding sequence of *M. quinquemaculata CYP6B46*
		Rev- TCAATTCGCTTGCGTAGGT	
10	*MqCYP6B45*	For- GATCAAAGATTTCGACGTGTTCAT	Amplification of partial coding sequence of *M. quinquemaculata CYP6B45*
		Rev- ACTTTGAGAGGGAAGATTGAAA	

### Real time quantitative PCR

Transcript levels of *Ubiquitin*, *CYP6B46*, *CYP6B45*, *BG1* and *BG2* were determined by qRT–PCR conducted in Mx3005P Multiplex qPCR system (Stratagene) using qRT–PCR SYBR Green I kit (Eurogentec). All the primer pairs (Table 2) used in qRT–PCR were designed using Primer3 software version 4.0 (http://primer3.ut.ee/) and were commercially synthesized (Sigma-Aldrich). The PCR reactions were performed in final volume of 10 μl containing the 2× qRT-PCR SYBR Green master mix (Eurogentec), 1 μl of primers (5 μM) and 4 μl of ten times diluted synthesized cDNA. Standard dilutions of pooled cDNA from at least five biological replicates were used for external standards. The following conditions were used for qPCR: initial denaturation step at 95 °C for 30s, followed by 40 cycles of amplification at 95 °C for 30s and 60 °C for 1 min, with a final extension step of 95 °C for 30s. To check the specificity of reactions, melt curve analysis was performed at 60–95 °C after 40 cycles and the PCR efficiency of each gene was tested (Additional file 1: Table S1). Relative quantification of transcripts was carried out by the comparative D cycle threshold method using external standard curves as previously standardized [19, 56]. *Mq*Ubiquitin levels were used as internal controls to normalize the transcript levels of target genes.

### Sequence similarity

*M. sexta’s CYP6B46* and *BG1* coding sequences used to generate PMRi lines with corresponding homologous coding sequences of *M. quinquemaculata* were aligned and sequence similarities were determined using nucleotide BLAST (www.ncbi.nlm.nih.gov). Aligned regions were scrutinized for continuous stretches >21 nt. In a similar way, *Mq*CYP6B46 and *Mq*BG1 partial coding sequences were aligned with potential off-target genes to determine their sequence similarity.

### Statistical analysis

All statistical analyses were performed with StatView version 5 (SAS Institute Inc.). All transcript levels were analyzed by one-way analysis of variance and the statistical significance (*P* ≤ 0.05) was determined by Fisher’s least significant difference post hoc tests.

## Additional file

Additional file 1: Table S1. Name, accession number, slope, efficiency and R^2^ of Mq target genes. (DOCX 13 kb)

## Abbreviations

BG1: β-glucosidase1
Bt: *Bacillus thuringiensis*
CPB: Colorado potato beetle
CYPs: Cytochrome P450 monooxygenases
dsRNA: double-stranded RNA
EV: Empty vector
HGL-DTG: 17-hydroxygeranyllinalool diterpene glycosides
ir: inverted repeat
irGGPPS: inverted repeat *geranylgeranyl pyrophosphate synthase*
irPMT: inverted repeat *putrescine N-methyl transferase*
Mq: *Manduca quinquemaculata*
Ms.: *Manduca sexta*
PMRi: Plant-mediated RNA interference
RNAi: RNA interference
