# Supplementary material for: Plant-mediated RNAi silences midgut-expressed genes in congeneric lepidopteran insects in nature
Source: BMC Plant Biol. 2017 Nov 13;17:199. doi: 10.1186/s12870-017-1149-5 (PMC5683459; doi:10.1186/s12870-017-1149-5)
Supplement: Additional file 1: Table S1. — Name, accession number, slope, efficiency and R2 of Mq target genes. (DOCX 13 kb) [file 12870_2017_1149_MOESM1_ESM.docx]

**Table S1. Name, accession number, slope, efficiency and R^2^ of Mq target genes.**

| **No** | **Name** | **Accession number** | **Slope** | **Efficiency (%)** | **R^2^** |
| --- | --- | --- | --- | --- | --- |
| 1 | *MqCYP6B46* | KX074015 | -3.3834 | > 97 | 0.999 |
| 2 | *MqBG1* | KX074013 | -3.3818 | > 97 | 0.998 |
| 3 | *MqCYP6B45* | KX074014 | -3.4947 | > 93 | 0.998 |
| 4 | *MqBG2* | KX074012 | -3.4681 | > 94 | 1 |
